# Clinical modifiers of the association between type 1 diabetes and dementia incidence

**DOI:** 10.1002/alz.71799

**Published:** 2026-09-20

**Authors:** Anna M. Pederson, Peter Buto, Scott C. Zimmerman, Mabeline Velez, Kendra D. Sims, Audrey R. Murchland, Jingxuan Wang, M. Maria Glymour, Nicole L. Spartano, Jennifer Weuve, Paola Gilsanz, Felicia Chi, Rachel A. Whitmer, Alana T. Brennan

**Affiliations:** ^1^ Department of Epidemiology and Biostatistics University of California San Francisco San Francisco California USA; ^2^ Department of Epidemiology Boston University School of Public Health Boston Massachusetts USA; ^3^ Department of Epidemiology Harvard T.H. Chan School of Public Health Boston Massachusetts USA; ^4^ Chobanian and Avedisian School of Medicine Boston University Boston Massachusetts USA; ^5^ Division of Research Kaiser Permanente Northern California Pleasanton California USA; ^6^ Department of Public Health Sciences University of California Davis California USA; ^7^ Department of Global Health Boston University School of Public Health Boston Massachusetts USA

**Keywords:** cardiovascular disease, dementia, diabetes complications, mental health, type 1 diabetes

## Abstract

**INTRODUCTION:**

Type 1 diabetes mellitus (T1DM) increases dementia risk. Understanding clinical modifiers of that effect could inform prevention.

**METHODS:**

We analyzed data from All of Us. Participants were aged ≥50 years with T1DM (*n* = 5,442) or no diabetes (*n* = 226,819).

**RESULTS:**

Among 232,261 participants (mean [SD] age 64.5 [9.0] years; 57.3% women), 2.3% had a T1DM diagnosis. Among those without diabetes, each additional comorbidity corresponded to a dementia hazard ratio (HR) of 1.22 (95% CI: 1.19, 1.26). The dementia HR among participants with both T1DM and depression, compared with having neither, was pronounced (HR: 5.47 [95% CI: 4.23, 7.08]) and consistent with the independent associations of T1DM alone (HR: 2.01 [95% CI:1.38, 2.92]) and depression alone (HR: 2.55 [95% CI: 2.22, 2.95]).

**DISCUSSION:**

Individuals who have both T1DM and depression are at especially high risk for dementia.

## Highlights

- In a large cohort with linked EHR information, T1DM and most diabetes‐related complications and comorbidities were independently associated with higher dementia incidence.
- For most conditions, co‐occurrence with T1DM did not confer excess dementia risk beyond the individual effects of T1DM and the comorbidity.
- Depression was a notable exception, showing evidence of a multiplicative effect with dementia risk when present alongside T1DM.
- These findings suggest that dementia risk in T1DM is largely driven by the independent effects of common comorbidities, highlighting depression as a potential target for risk reduction.

## 1 BACKGROUND

The strong association between type 1 diabetes mellitus (T1DM) and dementia is not well understood and in some studies appears to be greater than that observed for type 2 diabetes mellitus (T2DM).^1^, ^2^, ^3^ The limited available evidence suggests this risk may be amplified by co‐occurring conditions, such as cardiovascular disease (CVD) and depression, reflecting the earlier onset of T1DM and longer duration of complications.^4^, ^5^, ^6^ Identifying such modifiers could clarify mechanisms and inform care for people with T1DM.

T1DM may affect dementia risk through multiple pathways, including diabetes‐related complications, cardiovascular and metabolic comorbidities, and behavioral or mental health factors. Established dementia risk factors such as hypertension, stroke, chronic kidney disease (CKD), neuropathy, and hypoglycemia may contribute, though findings for retinopathy are mixed.^7^, ^8^, ^9^, ^10^, ^11^, ^12^, ^13^, ^14^ Depression, common among individuals with T1DM, may interact with glycemic control, creating a cycle of poorer self‐care and further complications.^4^ These mechanisms may be particularly relevant given that complications often emerge earlier in life among those with T1DM.^15^

Despite these plausible pathways, existing studies are few and typically underpowered, with the largest including fewer than 4000 individuals with T1DM.^4^, ^8^ Using data from a large electronic health record (EHR) cohort, we evaluated whether comorbidities and complications modified the association between T1DM and incident dementia.

## 2 METHODS

### 2.1 Study sample

The All of Us (AoU) Research Program is a cohort of US adults ≥ 18 years recruited through academic institutions and volunteer‐based enrollment. Participants completed surveys on sociodemographics, lifestyle, and health, and many consented to link EHRs.^16^, ^17^ Analyses were restricted to participants ≥50 years at baseline with linked EHRs (Figure S1). Follow‐up was available through October 10, 2023.

### 2.2 Assessment of T1DM

We used a validated algorithm to classify participants as having T1DM if they had at least one EHR encounter with a T1DM diagnostic code.^3^ Those without diabetes codes were classified as not having diabetes; individuals with T2DM but not T1DM were excluded.

### 2.3 Modifiers

Baseline comorbidities and complications were identified from EHRs and grouped into three conceptual domains: (1) diabetes complications and eye diseases (glaucoma, cataracts, retinopathy, neuropathy, hyper/hypoglycemic events); (2) cardiovascular and metabolic comorbidities (hypertension, dyslipidemia, obesity, myocardial infarction [MI], stroke, heart failure [HF], CKD); and (3) mental health conditions (alcohol use disorder [AUD], anxiety, and depression) (Table S1). Participants were classified as having a condition if at least one diagnostic code was present before the baseline survey. Obesity was additionally defined using measured body mass index (BMI) ≥ 30 kg/m^2^.^18^ Each condition was examined individually, and we additionally constructed four composite measures reflecting potential pathways linking comorbidity burden to dementia risk: (1) diabetes complications and eye diseases (range 0 to 6); (2) cardiovascular and metabolic comorbidities (range 0 to 7); (3) mental health conditions (range 0 to 3); and (4) overall comorbidity burden, defined as the total number of conditions across domains 1 to 3 (range 0 to 15).

Nearly all people with T1DM who developed dementia had hypertension or dyslipidemia at enrollment. Consequently, we did not estimate separate models for these comorbidities, as AoU reporting guidelines restrict reporting on samples of <20 participants.

### 2.4 Assessment of dementia

Dementia was defined as Alzheimer's disease (AD), vascular dementia, or dementia of unknown etiology, as done previously.^3^ Incident cases were identified from the first ICD‐9, ICD‐10, or SNOMED code after baseline. Participants with a prior dementia diagnosis were excluded, and follow‐up was censored at death or the last EHR encounter recorded in the data (October 10, 2023).

### 2.5 Covariates

We estimated the association between T1DM and incident dementia using models that adjusted for demographic confounding variables.^3^ All covariates were self‐reported at baseline and included age, gender (man/woman, self‐reported in the AoU “The Basics” survey using the “gender” variable, with only responses labeled “male” or “female” included), race and ethnicity (non‐Hispanic White, Hispanic/Latino, or non‐Hispanic Other), educational attainment (less than high school, high school, some college, or college and above), and household income (divided by the square root of household size).

In sensitivity analyses, we additionally adjusted models for smoking history (never smoked or ever smoking at least 100 cigarettes in your life), alcohol use (never, past, low use, and high use, based on the National Institute on Alcohol Abuse and Alcoholism [NIAAA] risk categories that account for frequency, quantity, and gender), and health insurance status to account for potential differences in healthcare access (yes/no).

### 2.6 Statistical analysis

We summarized baseline demographic and clinical characteristics of participants with and without T1DM. We fitted Cox proportional hazards models, using time since baseline as the time scale, to estimate hazard ratios (HRs) for the association between T1DM versus no diabetes diagnosis (T2DM excluded) and the risk of dementia. For each comorbidity, we fit models including T1DM, the comorbidity, and their interaction to estimate effect modification. We estimated two models: (1) demographic adjusted models, which adjusted for age, gender, race and ethnicity, education, and household income; and (2) a sensitivity analysis that additionally adjusted for smoking history, alcohol use, and health insurance status (maximally adjusted).

Effect modification was assessed on a multiplicative scale, where the interaction term estimates how much the combined effect of T1DM and the comorbidity exceeds (interaction HR > 1), equals (interaction HR = 1), or falls below (interaction HR < 1) the product of their individual effects. We generated three distinct estimates: (1) the effect of each modifier among people with T1DM; (2) whether the effect of the co‐occurrence of T1DM and each modifier was greater or less than what would be expected based on their individual HRs (i.e., deviation of the interaction term from 1); and (3) the joint association of co‐occurring T1DM and each modifier compared with individuals who had neither condition, estimated by combining the coefficients for T1DM, the modifier, and their interaction term on the log‐hazard scale (presented in Table S2).

To estimate the effect of each modifier among people with T1DM, combined HRs and 95% confidence intervals (CIs) were calculated as the sum of log‐HRs for each comorbidity and its interaction with T1DM.

$$\beta_{modifier}+\beta_{T1DM*modifier}$$

here $\beta_{modifier}$ is the coefficient for the comorbidity and $\beta_{T1DM*modifier}$ is the coefficient for its interaction with T1DM. The variance of this sum was derived from the model output's variance–covariance matrix:

$$\begin{array}{ccc} Var\left(\beta_{sum}\right) & = & Var\left(\beta_{modifier}\right)+Var\left(\beta_{T1DM*modifier}\right)\\ & & +\;2*\mathrm{Cov}\left(\beta_{modifier},\beta_{T1DM*modifier}\right), \end{array}$$

and the 95% CI was calculated by exponentiating the sum of the coefficients ± 1.96 times the square root of the sum of the variance:

$$95\%\;CI=exp[\left(\beta_{modifier}+\beta_{T1DM*modifier}\right)\;\pm \;\sqrt{Var\left(\beta_{sum}\right)}$$

To assess additive interaction, we estimated the relative excess risk due to interaction (RERI) for each T1DM‐comorbidity pair using the interaction package, with confidence intervals estimated using the delta method (Table S3).^19^, ^20^

**RESEARCH IN CONTEXT**

1. **Systematic review**: The authors reviewed the existing literature using databases (e.g., PubMed) and relevant abstracts. Prior studies reported an elevated risk of dementia among individuals with T1DM; however, evidence on the clinical pathways underlying this association remains limited. Few studies have systematically examined diabetes‐related complications and common comorbidities as potential modifiers of dementia risk in this population.
2. **Interpretation**: T1DM and most diabetes‐related complications and comorbidities were independently associated with increased dementia incidence. Depression was a notable exception, showing evidence of a multiplicative effect with dementia risk when co‐occurring with T1DM. These findings suggest that dementia risk in T1DM is largely driven by the independent effects of common comorbidities, with depression representing a potentially distinct and clinically important pathway.
3. **Future directions**: Future research should clarify mechanisms linking depression, T1DM, and dementia and evaluate whether the prevention and treatment of comorbidities reduce dementia risk.

The AoU Research Program protocol was approved by its Institutional Review Board (IRB) prior to enrollment. All participants provided informed consent, and data accessed through the secure Research Workbench are de‐identified. The Boston University Medical Campus IRB waived approval.

## 3 RESULTS

In our sample of 232,261 participants, 5,442 (2.3%) were classified as having T1DM and are the primary group of interest. Over a mean of 2.25 years of follow‐up, 1,416 (0.61%) participants developed dementia. At baseline, the mean age was 64.54 years (SD 8.98), 57.3% were female, and 50.0% had at least a college degree (Table 1). Participants had, on average, 1.69 total comorbidities (SD 2.08). This included a mean of 0.45 (SD 0.84) diabetes complications or eye conditions, 0.88 (SD 1.12) other CVD and metabolic comorbidities, and 0.34 (SD 0.70) mental health conditions.

**TABLE 1:** Characteristics of analytic sample, by diabetes status.

	All (*n* = 232,261)	Type 1 DM (*n* = 5,442)	No Diabetes (*n* = 226,819)
Age (years), mean (SD)	64.54 (8.98)	65.11 (8.72)	64.53 (8.99)
Woman, *n* (%)	133,038 (57.3%)	2741 (50.4%)	130,297 (57.4%)
Race and ethnicity			
NH White	145,849 (62.8%)	2597 (47.7%)	143,252 (63.2%)
Hispanic/Latino	28,281 (12.2%)	1030 (18.9%)	27,251 (12%)
NH other	58,131 (25%)	1815 (33.4%)	56,316 (24.8%)
Educational attainment			
Advanced degree	61,580 (26.5%)	825 (15.2%)	60,755 (26.8%)
College graduate	54,670 (23.5%)	1059 (19.5%)	53,611 (23.6%)
Some college	57,001 (24.5%)	1590 (29.2%)	55,411 (24.4%)
High school or GED	35,733 (15.4%)	1073 (19.7%)	34,660 (15.3%)
Less than high school	17,403 (7.5%)	739 (13.6%)	16,664 (7.3%)
BMI (kg/m^2^)	29.38 (6.94)	32.05 (7.43)	29.3 (6.91)
Income (divided by square root of household size)	60,363.86 (50981.14)	40,387.87 (41412.61)	60,808.77 (51085.14)
Ever smoked (100 cigarettes lifetime)	98,824 (42.5%)	2474 (45.4%)	96,350 (42.5%)
Glaucoma	12,837 (5.5%)	1375 (25.3%)	11,462 (5.1%)
Retinopathy	2959 (1.3%)	1325 (24.3%)	1634 (0.7%)
Cataracts	27,825 (12%)	2730 (50.2%)	25,095 (11.1%)
Neuropathy	49,185 (21.2%)	4092 (75.2%)	45,093 (19.9%)
Heart failure	6414 (2.8%)	1379 (25.3%)	5035 (2.2%)
Stroke	6982 (3%)	937 (17.2%)	6045 (2.7%)
Myocardial infarction	6546 (2.8%)	1080 (19.8%)	5466 (2.4%)
Chronic kidney disease	11,471 (4.9%)	2255 (41.4%)	9216 (4.1%)
Obesity	45,980 (19.8%)	1994 (36.6%)	43,986 (19.4%)
Hyperglycemic event	9533 (4.1%)	3085 (56.7%)	6448 (2.8%)
Hypoglycemic event	2291 (1%)	895 (16.4%)	1396 (0.6%)
Alcohol use disorder	9038 (3.9%)	664 (12.2%)	8374 (3.7%)
Anxiety	31,355 (13.5%)	2037 (37.4%)	29,318 (12.9%)
Depression	38,284 (16.5%)	2865 (52.6%)	35,419 (15.6%)
Diabetes complications and eye diseases^a^	0.45 (0.84)	2.48 (1.41)	0.40 (0.75)
Other vascular and metabolic comorbidities^b^	0.88 (1.12)	3.13 (1.50)	0.83 (1.05)
Mental health conditions^c^	0.34 (0.70)	1.02 (0.97)	0.32 (0.69)
Sum of overall comorbiditiesa^d^	1.69 (2.08)	6.65 (2.76)	1.57 (1.91)

^a^ Diabetes complications and eye diseases: total number of diabetes‐related complications including glaucoma, cataracts, retinopathy, neuropathy, and hyper‐ and hypoglycemic events.

^b^ Other vascular and metabolic comorbidities: total number of vascular and metabolic comorbidities, including dyslipidemia, hypertension, stroke, myocardial infarction, congestive heart failure, obesity, and chronic kidney disease.

^c^ Mental health conditions: count of alcohol use disorder, anxiety, and depression.

^d^ Sum of overall comorbidities: total number of comorbidities present at baseline across all comorbidities listed in groups a, b, and c.

Table 2 presents HRs and 95% CIs for dementia risk by comorbidity among individuals with T1DM from demographically adjusted models (adjusted for age, gender, race and ethnicity, education, and household income). Within the T1DM group, CKD (HR: 1.77 [95% CI: 1.18, 2.65]), anxiety (HR: 1.70 [95% CI: 1.14, 2.53]), and depression (HR: 2.72 [95% CI: 1.76, 4.20]) were associated with higher dementia incidence. Individuals with co‐occurring T1DM and these conditions had markedly elevated risk (e.g., T1DM + depression: HR: 5.47 [95% CI 4.23, 7.08]) compared with those with neither (Table 2, Table S2). Estimated comorbidity effects among people with T1DM ranged from HR 1.10 (glaucoma) to HR 2.72 (depression), though CIs included 1 for most comorbidities (Table 2, Table S2).

**TABLE 2:** Dementia hazard ratios for each comorbidity or complication (potential modifiers).

Modifier	Dementia cases/among T1D individuals *without* the modifier	Dementia cases/among T1D individuals *with* the modifier	Dementia hazard ratio for comorbidities among individuals with T1D (95% confidence interval)	Dementia hazard ratio for comorbidities among individuals with no DM (95% confidence interval)
Glaucoma (ref: no glaucoma)	103/3964	41/1334	1.10 (0.72, 1.68)	1.30 (1.07, 1.58)
Cataracts (ref: no cataracts)	49/2663	95/2635	1.22 (0.81, 1.86)	1.25 (1.07, 1.45)
Retinopathy (ref: no retinopathy)	101/4016	43/1282	1.31 (0.86, 2.00)	1.48 (0.96, 2.29)
Neuropathy (ref: no neuropathy)	31/1319	113/3979	1.17 (0.71, 1.91)	1.72 (1.51, 1.98)
Hyperglycemic event (ref: no hyperglycemic event)	59/2298	85/3000	1.45 (0.97, 2.17)	1.54 (1.18, 2.03)
Hypoglycemic event (ref: no hypoglycemic event)	113/4434	31/864	1.34 (0.82, 2.19)	3.71 (2.45, 5.62)
Heart failure (ref: no heart failure)	96/3967	48/1331	1.13 (0.74, 1.72)	2.00 (1.59, 2.51)
Stroke (ref: no stroke)	98/4407	46/891	1.46 (0.94, 2.26)	2.34 (1.91, 2.87)
Myocardial infarction (ref: no myocardial infarction)	106/4256	38/1042	1.14 (0.72, 1.80)	1.91 (1.51, 1.98)
Chronic kidney disease (ref: no chronic kidney disease)	66/3121	78/2177	1.77 (1.18, 2.65)	1.61 (1.32, 1.97)
Obesity (ref: no obesity)	89/3359	55/1939	1.01 (0.67, 1.52)	1.09 (0.91, 1.30)
Alcohol use disorder (ref: no alcohol use disorder)	122/4656	22/642	1.37 (0.78, 2.43)	1.72 (1.30, 2.28)
Anxiety (ref: no anxiety)	85/3320	59/1978	1.70 (1.14, 2.53)	2.29 (1.97, 2.67)
Depression (ref: no depression)	47/2530	97/2768	2.72 (1.76, 4.20)	2.55 (2.22, 2.95)
Diabetes complications and eye diseases^a^, per complication			1.16 (1.00, 1.34)	1.27 (1.19, 1.35)
Other vascular and metabolic comorbidities^b^, per comorbidity			1.17 (1.03, 1.34)	1.36 (1.30, 1.43)
Mental health conditions^c^, per condition			1.53 (1.25, 1.88)	1.66 (1.54, 1.79)
Sum of overall comorbidities^d^, per comorbidity			1.15 (1.07, 1.24)	1.22 (1.19, 1.26)

*Note*: All hazard ratios are adjusted for age, gender, race and ethnicity, educational attainment, and household income. Hazard ratios for each comorbidity among individuals with T1DM were derived by multiplying the main effect of the comorbidity by the interaction between the comorbidity and T1DM, as reported in Table S1.

^a^ Diabetes complications and eye diseases: total number of diabetes‐related complications including glaucoma, cataracts, retinopathy, neuropathy, and hyper‐ and hypoglycemic events.

^b^ Other vascular and metabolic comorbidities: total number of CVD and metabolic comorbidities, including dyslipidemia, hypertension, stroke, myocardial infarction, heart failure, obesity, and chronic kidney disease.

^c^ Mental health conditions: count of alcohol use disorder, anxiety, and depression.

^d^ Sum of overall comorbidities: total number of comorbidities present at baseline across all comorbidities listed in groups denoted by a, b, and c.

**FIGURE 1:**
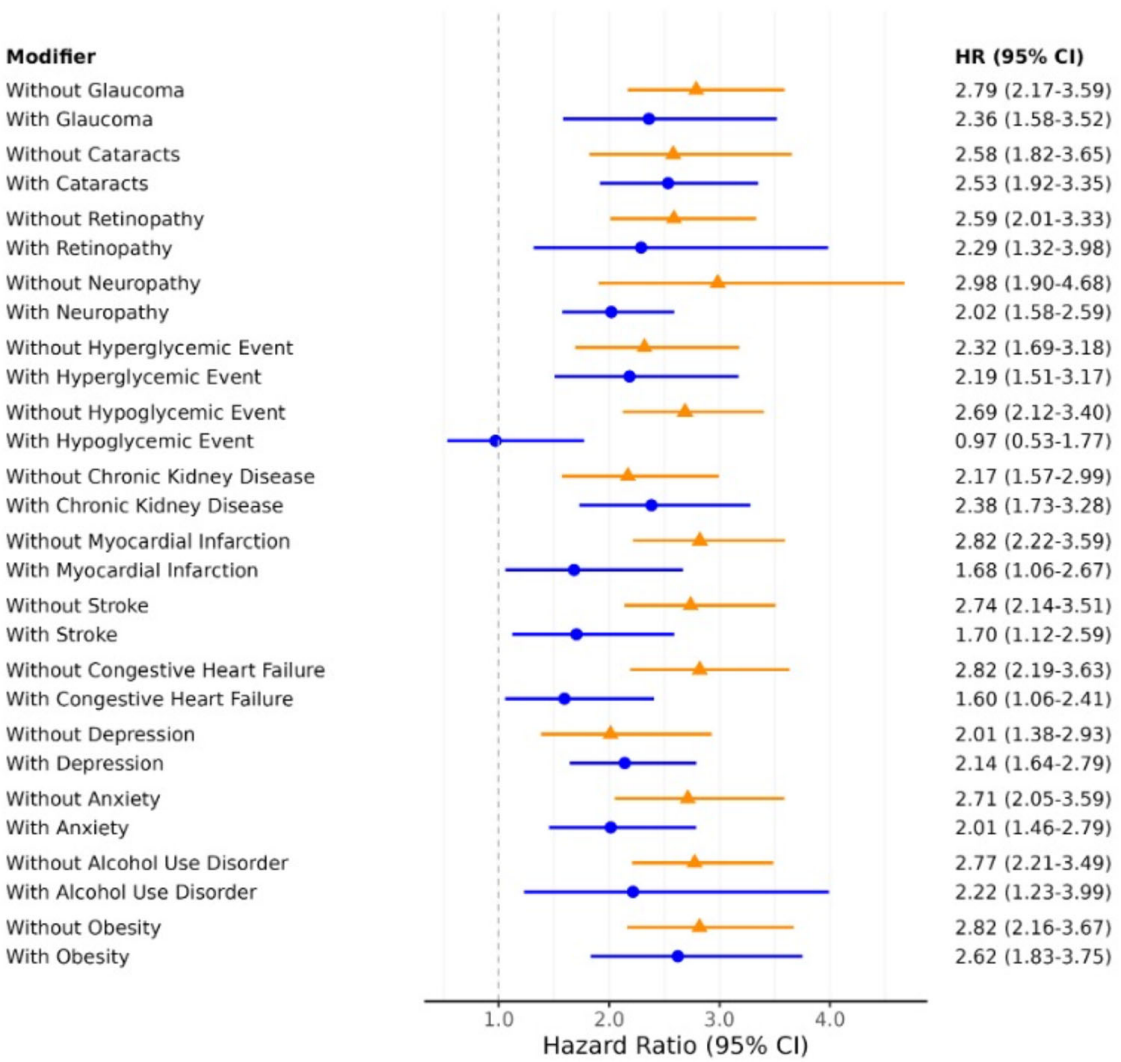
Associations and 95% confidence intervals of type 1 diabetes (T1DM) with dementia risk by modifier status. Estimates in orange show the main effect of T1DM in individuals without the modifier. Estimates in blue show the main effect of T1DM in individuals with the modifier. All models were adjusted for age, race and ethnicity, gender, education, and income.

Although point estimates for both T1DM and each comorbidity were associated with elevated dementia hazard (i.e., HR > 1), the combined effects of T1DM and most comorbidities were less than multiplicative. For example, the T1DM–HF interaction was <1.0, suggesting that the estimated effect of T1DM was smaller among individuals with HF (HR: 1.60 [95% CI: 1.06, 2.41]) compared to the estimated effect of T1DM among those without HF (HR 2.82 [95% CI: 2.18, 3.63]) (Figure 1). Interactions were largely below 1 with wide CIs, with smaller relative effects of T1DM among those with MI (HR: 0.56 [95% CI: 0.35, 0.99]), stroke (HR: 0.62 [95% CI: 0.38, 1.00]), and hypoglycemic events (HR: 0.36 [95% CI: 0.19, 0.68]) (Table S2).

Additionally, comorbidity burden was positively associated with dementia incidence. For example, among people without T1DM, each additional comorbidity in the sum of the overall count was associated with a 22% increase in dementia incidence (HR: 1.22 [95% CI: 1.19, 1.26]). Similar patterns were observed for each additional vascular and metabolic comorbidity (HR: 1.36 [95% CI: 1.30, 1.43]), diabetes complications or eye condition (HR: 1.27 [95% CI: 1.19, 1.35]), and mental health comorbidity (HR: 1.66 [95% CI: 1.54, 1.79]) (Table S2).

In sensitivity analyses, results were robust to further adjustment for smoking history, alcohol use, and health insurance status (Table S2).

RERI estimates were close to 0 with wide confidence intervals for most comorbidities, offering limited evidence of synergistic interaction on an additive scale. However, depression showed significant positive additive interaction in both the demographic‐adjusted (RERI 1.91 [95% CI: 0.38, 3.44]) and maximally adjusted (RERI 1.63 [95% CI: 0.16, 3.10]) models, indicating that the combined effect of T1DM and depression on dementia risk exceeded the sum of their individual effects. A similar pattern was observed for the mental health conditions domain (demographic‐adjusted RERI: 0.59 [95% CI: 0.11, 1.07]; maximally adjusted RERI: 0.54 [95% CI: 0.05, 1.03]) (Table S3).

## 4 DISCUSSION

This is among the first large EHR‐based studies to examine a broad range of clinical and behavioral comorbidities as modifiers of the association between T1DM and incident dementia. T1DM and most comorbidities were independently associated with higher dementia incidence; however, interaction analyses generally showed no excess risk beyond their individual effects. An exception was depression, for which co‐occurring T1DM and depression showed evidence of a multiplicative association with dementia incidence.

Although mechanistic interpretations of interaction are speculative, deviations from multiplicativity may reflect partial overlap of causal pathways, such that additional exposure contributes less incremental risk. In contrast, interaction terms near 1 on the multiplicative scale, as observed for cataracts, hyperglycemic events, CKD, and depression, are compatible with approximately proportional effects across strata and do not suggest meaningful departure from multiplicativity.

On the additive scale, however, depression showed evidence of synergistic interaction, suggesting that the combined effect of T1DM and depression on dementia risk exceeded the sum of their individual effects. This additive interaction implies that while the relative effects of T1DM and depression are approximately proportional, their absolute combined effect represents a greater burden than either condition alone, which has relevance for identifying high‐risk subgroups in clinical practice.

Our study has several strengths, including a validated EHR‐based T1DM algorithm, a large sample including 5,442 individuals with T1DM, and detailed ascertainment of comorbidities often underreported in surveys.^3^

## 5 LIMITATIONS

T1DM and comorbidities may be misclassified in EHRs, and even modest misclassification could influence estimates given that T1DM is relatively rare. The observed T1DM prevalence of 2.3% in our cohort is somewhat higher than population estimates for older adults, which may reflect some inclusion of atypical or mixed diabetes phenotypes. Our T1DM classification follows a validated algorithm; however, the classification threshold may not be optimal for all analytic purposes and may depend on the research question at hand.^3^ Comorbidities may also be under‐captured if care occurred outside contributing systems or developed after baseline, potentially attenuating associations with dementia risk.

Because AoU is a convenience sample drawn largely from academic institutions and partnering clinics, participants' EHR data may be incomplete and miss care received across multiple health systems. The timing of T1DM diagnosis was largely unavailable: oanly a subset of participants completed the question on age at diagnosis, which included only three age categories. Consequently, we were unable to estimate T1DM duration. Survival to age 50 may be a marker of better‐managed or less severe T1DM, and life expectancy among those with T1DM has improved substantially over time. Life expectancy for those diagnosed between 1965 and 1980 was approximately 15 years greater than for those diagnosed 1950 to 1964 (68.8 [95% CI: 64.7, 72.8] vs 53.4 [50.8, 56.0] years, respectively).^21^ Because longer disease duration may increase dementia risk through cumulative vascular and metabolic burden, future studies with better onset data should examine this relationship.

Temporality cannot be fully established, as comorbidity definitions did not distinguish long‐standing conditions from new diagnoses, and symptoms of dementia often begin years prior to its diagnosis. This concern is greatest for conditions that may emerge shortly before dementia onset, such as late‐life depression and acute metabolic events. For most comorbidities examined, though, diagnoses likely substantially predate enrollment, reducing the plausibility of reverse causation. For example, among participants with depression at enrollment, 85% had their first recorded diagnosis *at least* 1 year prior and 50% at least 5 years prior; because records were not available going back farther for many participants, this suggests depression onset substantially preceded enrollment for most participants. Nevertheless, we cannot determine whether diagnoses originated in youth, midlife, or late life, and the distinct pathways through which depression at different life stages may influence dementia risk cannot be disentangled here.

Follow‐up from enrollment was relatively short (mean 2.25 years; maximum 6.34 years) relative to the long latency period of dementia. A proportion of cases identified during follow‐up may represent prevalent but undiagnosed dementia at baseline, particularly given reliance on EHR‐based diagnoses. Although mild cognitive impairment (MCI) represents an earlier manifestation of cognitive decline, MCI diagnoses are inconsistently captured in EHRs and may be even more influenced by patterns of healthcare utilization than dementia diagnoses. Given this, we focused on dementia as a more reliably ascertained outcome.

Because dementia diagnoses in EHR data depend on clinical recognition and documentation, observed associations may partly reflect differential intensity in healthcare utilization rather than true differences in incidence. Individuals with T1DM had higher encounter rates than those without diabetes, which increased opportunities for dementia to be diagnosed and may have resulted in overestimation of the association between T1DM and dementia. For example, 89% of participants with T1DM had three or more encounters in the first year after baseline, compared to only 45% of participants without diabetes. The proportion with no recorded encounter for 448 days after baseline, however, was very low for both groups (T1DM: 0.09%, no diabetes: 0.15%), suggesting that healthcare engagement was high across groups regardless of diabetes status (Table S4). Comparisons of dementia risk across comorbidity groups within the T1DM population are less susceptible to this bias, as healthcare engagement is uniformly high. Sensitivity analyses restricted to participants with high healthcare engagement in a prior analysis of this cohort yielded attenuated but still markedly elevated hazard ratios for T1DM, supporting the robustness of this association.^3^

Additionally, because we only used participants who self‐reported their gender identity as “man” or “woman,” our analyses do not capture non‐binary or other gender identities, which limits generalizability to gender‐minority populations. Finally, although we focused on depression and anxiety given their high prevalence and established associations with cognitive decline, other potentially pertinent psychiatric conditions such as bipolar disorder and eating disorders were too rare within the analytic sample to evaluate.

Overall, our results suggest that nearly every comorbidity was associated with increased dementia incidence among people with T1DM, with particularly large excesses associated with CKD, anxiety, and depression. These increases were generally not more than what would be expected if T1DM and the comorbidity independently influence dementia risk.

## CONFLICT OF INTEREST STATEMENT

The authors declare no conflicts of interest. Author disclosures are available in the Supporting Information.

## CONSENT STATEMENT

The AoU Research Program protocol was approved by its IRB prior to enrollment. All participants provided informed consent, and data accessed through the secure Research Workbench are de‐identified. The Boston University Medical Campus IRB waived approval.

## Supporting information



Supporting Information

Supporting Information
