# Supplementary material for: Clinical modifiers of the association between type 1 diabetes and dementia incidence
Source: Alzheimers Dement. 2026 Sep 20;22(9):e71799. doi: 10.1002/alz.71799 (PMC13590933; doi:10.1002/alz.71799)
Supplement: Supplementary file 2 [file ALZ-22-e71799-s002.docx]

#### Supplemental Table 1. Concept IDs Used for Each Comorbidity

| **Comorbidity** | Concept ID(s) | Notes |
| --- | --- | --- |
| Glaucoma | 437541, 44826578, 1568761, 441561, 44828911, 156769, 441284, 44834658, 1568768, 435543, 44819624, 1568792, 425262 | Includes descendants and all mapped codes |
| Cataracts | 1568646, 44830011, 375545, 44828918, 44830014, 44837042, 44828917, 35207548, 44823055, 44824186, 44830013, 44837041, 1568667, 44828913, 44833484, 1568670, 44831167, 44827724, 44830010, 44826580, 44828915, 44837043, 35207544, 44834663, 44831166, 44820797, 44835867, 44831165, 44819629, 44828914, 44833483, 44819631, 44828916, 1568664, 44830012, 44827725, 35207545 | Includes descendants and all mapped codes |
| Retinopathy | 44831148, 44833466, 44827714, 45538261, 44820785, 44833465, 1568707, 44835858 | Includes descendants and all mapped codes |
| Neuropathy | 44827615, 44825331, 1568864, 35207451, 44822493, 44822492, 44826006, 44827706, 44828884, 35207477, 44829067, 4301699, 44827690, 44825349, 1568865, 44826563 | Includes descendants and all mapped codes |
| Hyperglycemic Event | [4214376](https://databrowser.researchallofus.org/ehr/conditions/4214376) | Includes descendants and all mapped codes |
| Hypoglycemic Event | 24609, 44831048, 35206889, 380688, 35206888, 44820686 | Includes descendants and all mapped codes |
| Heart Failure | 4023479, 44782655, 4242669, 4229440, 319835, 314378 | Includes descendants and all mapped codes |
| Stroke | 1569193, 1569190, 1569184, 35207403, 35207404, 35207405, 35207406, 35207407, 44820872, 44835946, 44820873, 44824253, 443454, 432923 | Includes descendants and all mapped codes |
| Myocardial Infarction | 4329847, 312327, [1326591](https://databrowser.researchallofus.org/ehr/conditions/1326591), [1569126](https://databrowser.researchallofus.org/ehr/conditions/1569126) | Includes descendants and all mapped codes |
| Chronic Kidney Disease | 46271022, 44830172, 1571486, 443614, 443601, 443597, 443612, 443611, 35209274, 35209275, 35209276, 725440, 725441, 725442, 35209277, 35209278, 44837191, 44830173, 44827888, 44820970, 44837192 | Includes descendants and all mapped codes |
| Alcohol Use Disorder | 44824125, 44826511, 433753, 44820730, 1568096, 45581404, 45600692, 45571707, 45538006, 1568097, 45576493, 45557154, 45576494, 1568099, 45576495, 725273, 725274, 725275, 44826518, 44829936, 45595845, 44828841, 725271, 435243, 1568100, 44832244, 45566777, 45562002, 45595847, 45566776, 45600695, 45562003, 45557155, 45571708, 45600696, 45591082, 45586189, 45586188, 45538007, 45571709, 45591084, 45605452, 45552431, 45566775, 45586190, 45533065, 45586187, 1568102, 45566781, 45542791, 45566780, 45547675, 1568104, 45591087, 45586192, 45562005, 1568103, 45566778, 45595851, 45552432, 45576497, 1568105, 45605456, 725276, 725278, 725277, 725280, 37402462, 37402461, 44826487, 44835774, 44824107, 44824108, 44832220, 44836956, 44819550, 44825305, 44819551, 44831100, 44831080 | Includes descendants and all mapped codes |
| Anxiety | 442077, 440374, 4304010, 4152371 | Includes descendants and all mapped codes |

#### Supplemental Table 2. Dementia Hazard Ratios for Type 1 Diabetes, Modifiers, and Their Interaction

|  | Dementia hazard ratio (95% confidence interval), diabetes type (type 1 diabetes, no diabetes [referent]) | |
| --- | --- | --- |
|  | Demographic Adjusted | Fully Adjusted |
| Comorbidity |  |  |
|  | | |
| Type 1 Diabetes | 2.78 (2.16, 3.58) | 2.60 (2.00, 3.38) |
| Glaucoma | 1.30 (1.07, 1.58) | 1.29 (1.05, 1.58) |
| Glaucoma: T1DM Interaction | 0.84 (0.53, 1.35) | 0.88 (0.54, 1.41) |
| Co-occurring T1DM and Glaucoma | 3.08 (2.14, 4.43) | 2.93 (2.02, 4.25) |
|  | | |
| Type 1 Diabetes | 2.58 (1.82, 3.65) | 2.41 (1.68, 3.45) |
| Cataracts | 1.25 (1.07, 1.45) | 1.25 (1.07, 1.46) |
| Cataracts: T1DM Interaction | 0.98 (0.63, 1.52) | 0.99 (0.63, 1.57) |
| Co-occurring T1DM and Cataracts | 3.17 (2.44, 4.12) | 2.98 (2.28, 3.90) |
|  | | |
| Type 1 Diabetes | 2.58 (2.00, 3.33) | 2.42 (1.86, 3.14) |
| Retinopathy | 1.48 (0.96, 2.29) | 1.42 (0.90. 2.24) |
| Retinopathy: T1DM Interaction | 0.88 (0.48, 1.62) | 0.94 (0.50, 1.75) |
| Co-occurring T1DM and Retinopathy | 3.40 (2.37, 4.86) | 3.22 (2.24, 4.63) |
|  | | |
| Type 1 Diabetes | 2.98 (1.90, 4.67) | 2.60 (1.59, 4.23) |
| Neuropathy | 1.72 (1.51, 1.98) | 1.75 (1.52, 2.01) |
| Neuropathy: T1DM | 0.67 (0.40, 1.12) | 0.74 (0.43, 1.28) |
| Co-occurring T1DM and Neuropathy | 3.49 (2.74, 4.45) | 3.37 (2.63, 4.32) |
|  | | |
| Type 1 Diabetes | 2.39 (1.69, 3.17) | 2.24 (1.62, 3.08) |
| Hyperglycemic Event | 1.54 (1.18, 2.03) | 1.44 (1.08, 1.92) |
| Hyperglycemic Event: T1DM Interaction | 0.94 (0.58, 1.52) | 0.96 (0.58, 1.58) |
| Co-occurring T1DM and Hyperglycemic Event | 3.38 (2.57, 4.43) | 3.10 (2.34, 4.11) |
|  | | |
| Type 1 Diabetes | 2.68 (2.12, 3.40) | 2.57 (2.01, 3.27) |
| Hypoglycemic Event | 3.71 (2.45, 5.62) | 3.44 (2.23, 5.30) |
| Hypoglycemic Event: T1DM | 0.36 (0.19, 0.68) | 0.36 (0.18, 0.70) |
| Co-occurring T1DM and Hypoglycemic Event | 3.60 (2.31, 5.64) | 3.16 (1.98, 5.06) |
|  | | |
| Type 1 Diabetes | 2.82 (2.18, 3.63) | 2.59 (1.99, 3.37) |
| HF | 2.00 (1.59, 2.51) | 1.95 (1.54, 2.47) |
| HF: T1DM Interaction | 0.56 (0.35, 0.91) | 0.62 (0.38, 1.01) |
| Co-occurring T1DM and HF | 3.19 (2.23, 4.57) | 3.14 (2.19, 4.51) |
|  | | |
| Type 1 Diabetes | 2.73 (2.13, 3.50) | 2.53 (1.95, 3.27) |
| Stroke | 2.34 (1.91, 2.87) | 2.37 (1.92,2.91) |
| Stroke: T1DM Interaction | 0.62 (0.38, 1.00) | 0.66 (0.41, 1.07) |
| Co-occurring T1DM and Stroke | 4.00 (2.73, 5.86) | 3.95 (2.69, 5.79) |
|  | | |
| Type 1 Diabetes | 2.82 (2.21, 3.59) | 2.66 (2.07, 3.41) |
| MI | 1.91 (1.51, 1.98) | 1.93 (1.51, 2.46) |
| MI: T1DM Interaction | 0.56 (0.35, 0.99) | 0.59 (0.35, 1.00) |
| Co-occurring T1DM and MI | 3.21 (2.13, 4.85) | 3.03 (1.99, 4.60) |
|  | | |
| Type 1 Diabetes | 2.17 (1.57, 2.99) | 2.12 (1.53, 2.94) |
| CKD | 1.61 (1.32, 1.97) | 1.61 (1.31, 1.99) |
| CKD: T1DM Interaction | 1.09 (0.70, 1.71) | 1.03 (0.65, 1.63) |
| Co-occurring T1DM and CKD | 3.85 (2.94, 5.05) | 3.53 (2.66, 4.67) |
|  | | |
| Type 1 Diabetes | 2.81 (2.16, 3.67) | 2.60 (1.98, 3.42) |
| Obesity | 1.09 (0.91, 1.30) | 1.05 (0.87, 1.26) |
| Obesity: T1DM Interaction | 0.93 (0.59, 1.44) | 0.99 (0.63, 1.56) |
| Co-occurring T1DM and Obesity | 2.85 (2.04, 3.99) | 2.71 (1.92, 3.81) |
|  | | |
| Type 1 Diabetes | 2.77 (2.20, 3.48) | 2.72 (2.15, 3.43) |
| AUD | 1.72 (1.30, 2.28) | 1.72 (1.29, 2.28) |
| AUD: T1DM Interaction | 0.79 (0.42, 1.49) | 0.77 (0.40, 1.47) |
| Co-occurring T1DM and AUD | 3.82 (2.24, 6.53) | 3.58 (2.06, 6.24) |
|  | | |
| Type 1 Diabetes | 2.71 (2.05, 3.58) | 2.59 (1.95, 3.46) |
| Anxiety | 2.29 (1.97, 2.67) | 2.27 (1.95, 2.66) |
| Anxiety: T1DM Interaction | 0.74 (0.48, 1.13) | 0.73 (0.47, 1.13) |
| Co-occurring T1DM and Anxiety | 4.63 (3.39, 6.31) | 4.30 (3.11, 5.94) |
|  | | |
| Type 1 Diabetes | 2.01 (1.38, 2.92) | 1.94 (1.32, 2.85) |
| Depression | 2.55 (2.22, 2.95) | 2.51 (2.17, 2.91) |
| Depression: T1DM Interaction | 1.06 (0.67, 1.67) | 1.04 (0.65, 1.66) |
| Co-occurring T1DM and Depression | 5.47 (4.23, 7.08) | 5.06 (3.88, 6.60) |
|  | | |
| Type 1 Diabetes | 2.23 (1.40, 3.55) | 2.09 (1.29, 3.38) |
| Diabetes Complications and Eye Diseases | 1.27 (1.19, 1.35) | 1.27 (1.19, 1.35) |
| Diabetes Complications and Eye Disease: T1DM Interaction | 0.91 (0.78, 1.06) | 0.92 (0.78, 1.08) |
| Co-occurring T1DM and Diabetes Complications and Eye Diseases | 2.60 (1.84, 3.68) | 2.43 (1.70, 3.48) |
|  | | |
| Type 1 Diabetes | 2.56 (1.50, 4.38) | 2.32 (1.33, 4.05) |
| Vascular Comorbidities | 1.36 (1.30, 1.43) | 1.36 (1.30, 1.43) |
| Vascular Comorbidities: T1DM Interaction | 0.86 (0.75, 0.99) | 0.87 (0.76, 1.01) |
| Co-occurring T1DM and Vascular Comorbidities | 3.03 (1.99, 4.61) | 2.77 (1.79, 4.28) |
|  | | |
| Type 1 Diabetes | 2.32 (1.66, 3.26) | 2.31 (1.64, 3.25) |
| Mental Health Comorbidities | 1.66 (1.54, 1.79) | 1.67 (1.55, 1.80) |
| Mental Health Comorbidities: T1DM Interaction | 0.92 (0.74, 1.14) | 0.91 (0.73, 1.13) |
| Co-occurring T1DM and Mental Health Comorbidities | 3.58 (2.85, 4.50) | 3.52 (2.79, 4.43) |
|  | | |
| Type 1 Diabetes | 1.77 (0.95, 3.30) | 1.60 (0.84, 3.04) |
| Sum of Comorbidities | 1.22 (1.19, 1.26) | 1.23 (1.20, 1.26) |
| Sum of Comorbidities: T1DM Interaction | 0.94 (0.86, 1.01) | 0.95 (0.87, 1.02) |
| Co-occurring T1DM and Sum of Comorbidities | 2.05 (1.18, 3.57) | 1.86 (1.05, 3.30) |

Demographic models are adjusted for age, gender, race and ethnicity, educational attainment, and household income. Maximally adjusted models additionally adjust for smoking status, alcohol use, and health insurance status. [*] Diabetes complications and Eye Diseases: Total number of diabetes-related complications including glaucoma, cataracts, retinopathy, neuropathy, and hyper- and hypo- glycemic events. [†] Other vascular and metabolic comorbidities: Total number of vascular and metabolic comorbidities, including dyslipidemia, hypertension, stroke, myocardial infarction, congestive heart failure, obesity, and chronic kidney disease. [‡] Mental health conditions: Count of alcohol use disorder, anxiety, and depression. [§] Sum of overall comorbidities: Total number of comorbidities present at baseline across all comorbidities listed in groups [*], [†], and [‡].

Supplemental Table 3. Additive Interaction (RERI) Between Type 1 Diabetes and Comorbid Conditions on Incident Dementia Risk

|  | Demographic Adjusted | Maximally Adjusted |
| --- | --- | --- |
| Comorbidity |  |  |
| **Glaucoma** | -0.01 (-1.31, 1.28) | 0.05 (-1.20, 1.30) |
| **Cataracts** | 0.34 (-0.83, 1.51) | 0.32 (-0.81, 1.46) |
| **Retinopathy** | 0.33 (-1.15, 1.81) | 0.38 (-1.05, 1.81) |
| **Neuropathy** | -0.22 (-1.75, 1.31) | 0.04 (-1.42, 1.50) |
| **Hyperglycemic Event** | 0.52 (-0.68, 1.71) | 0.43 (-0.73, 1.58) |
| **Hypoglycemic Event** | -1.79 (-4.08, 0.49) | -1.83 (-4.00, 0.33) |
| **Heart Failure (HF)** | -0.63 (-2.00, 0.74) | -0.38 (-1.72, 0.97) |
| **Stroke** | -0.08 (-1.76, 1.59) | 0.05 (-1.60, 1.70) |
| **Myocardial Infarction (MI)** | -0.52 (-2.02, 0.99) | -0.54 (-2.00, 0.92) |
| **Chronic Kidney Disease (CKD)** | 1.07 (-0.16, 2.30) | 0.80 (-0.39, 1.99) |
| **Obesity** | -0.05 (-1.22, 1.12) | 0.06 (-1.06, 1.18) |
| **Alcohol Use Disorder (AUD)** | 0.33 (-1.82, 2.47) | 0.15 (-1.94, 2.24) |
| **Anxiety** | 0.62 (-0.96, 2.20) | 0.45 (-1.08, 1.98) |
| **Depression** | 1.91 (0.38, 3.44) | 1.63 (0.16, 3.10) |
| **Diabetes Complications and Eye Diseases*** | 0.09 (-0.15, 0.33) | 0.07 (-0.16, 0.31) |
| **Other Vascular and Metabolic Comorbidities**^†^ | 0.09 (-0.11, 0.30) | 0.08 (-0.10, 0.26) |
| **Mental Health Conditions**^‡^ | 0.59 (0.11, 1.07) | 0.54 (0.05, 1.03) |
| **Sum of Overall Comorbidities**^§^ | 0.05 (-0.01, 0.11) | 0.03 (-0.03, 0.09) |

Demographic models are adjusted for age, gender, race and ethnicity, educational attainment, and household income. Maximally adjusted models additionally adjust for smoking status, alcohol use, and health insurance status. [*] Diabetes complications and Eye Diseases: Total number of diabetes-related complications including glaucoma, cataracts, retinopathy, neuropathy, and hyper- and hypo- glycemic events. [†] Other vascular and metabolic comorbidities: Total number of vascular and metabolic comorbidities, including dyslipidemia, hypertension, stroke, myocardial infarction, congestive heart failure, obesity, and chronic kidney disease. [‡] Mental health conditions: Count of alcohol use disorder, anxiety, and depression. [§] Sum of overall comorbidities: Total number of comorbidities present at baseline across all comorbidities listed in groups [*], [†], and [‡].

Supplemental Table 4**.** Follow-up patterns by diabetes type

| Diabetes Type | % with no recorded encounter for 448 days after baseline | % with three or more encounters within the first year after baseline |
| --- | --- | --- |
| No Diabetes | 0.15 | 0.45 |
| Type 1 Diabetes | 0.09 | 0.89 |
